# Mechanistic Assessment of Cardiovascular State Informed by Vibroacoustic Sensors

**DOI:** 10.3390/s24072189

**Published:** 2024-03-29

**Authors:** Ali Zare, Emily Wittrup, Kayvan Najarian

**Affiliations:** 1 Department of Computational Medicine and Bioinformatics, University of Michigan, Ann Arbor, MI 48103, USA; 2 Department of Emergency Medicine, University of Michigan, Ann Arbor, MI 48103, USA; 3 Department of Electrical Engineering and Computer Science, University of Michigan, Ann Arbor, MI 48103, USA; 4 Michigan Institute for Data Science, University of Michigan, Ann Arbor, MI 48103, USA

**Keywords:** mechanistic model, cardiovascular, hemodynamics, vibroacoustics

## Abstract

Monitoring blood pressure, a parameter closely related to cardiovascular activity, can help predict imminent cardiovascular events. In this paper, a novel method is proposed to customize an existing mechanistic model of the cardiovascular system through feature extraction from cardiopulmonary acoustic signals to estimate blood pressure using artificial intelligence. As various factors, such as drug consumption, can alter the biomechanical properties of the cardiovascular system, the proposed method seeks to personalize the mechanistic model using information extracted from vibroacoustic sensors. Simulation results for the proposed approach are evaluated by calculating the error in blood pressure estimates compared to ground truth arterial line measurements, with the results showing promise for this method.

## 1. Introduction

Computational modeling of cardiovascular hemodynamics, although a challenging task due to the complex properties of various components in the cardiovascular system, can provide non-invasive, simulated blood flow monitoring, allowing for improved diagnosis of cardiovascular diseases. In Rosalia et al., a lumped-parameter Windkessel model of the cardiovascular system was developed that simulated the mechanistic parameters of the cardiovascular, pulmonary, abdominal, and upper body systems [1]. This allows for simulated blood pressure readings at various points of the cardiovascular system, including those close to locations where invasive blood pressure measurements are usually performed in practice, namely, cuff or arterial line locations.

Cardiopulmonary acoustic signals provide a rich source of information on cardiovascular health and have been used previously to label and detect abnormalities and calculate blood pressure, paving the way for the detection of cardiovascular disorders. In Guo et al., a deep learning approach has been presented to automatically annotate heart sound readings within label groups such as pitch and shape [2]. Abduh et al. used mel-frequency coefficients based on the fractional Fourier transform to distinguish normal cardiopulmonary acoustic signals from those that are abnormal using machine learning classifiers such as support vector machines and k-nearest neighbors [3]. Finally, in Chen et al., the $s_{1}$ and $s_{2}$ cardiopulmonary acoustic signal channels are separated and used to estimate the systolic and diastolic blood pressure values [4]. In addition, another potential and interesting application of the proposed approach is drug effect assessment. Monitoring blood pressure as a parameter related to cardiovascular activity can help assess the effect of a certain medication. As drugs such as nonaspirin nonsteroidal anti-inflammatory drugs can alter the biomechanical properties of the cardiovascular system, affecting an individual’s cardiopulmonary acoustic signals and blood pressure, a mechanistic model can be modified to reflect such changes and further personalized using vibroacoustic signals, assessing the effect of a specific drug on blood pressure [5].

This paper aims to combine the merits of a mechanistic cardiovascular model and vibroacoustic sensors to develop a semi-continuous blood pressure monitoring scheme for cardiovascular state assessment using a machine learning framework. In Section 2, the proposed methodology and algorithm are presented. Section 3 provides details about a case where vibroacoustic signals of the heart are preprocessed and used as input to the proposed approach. In Section 4, simulation results are presented and discussed. Finally, Section 5 provides concluding remarks along with suggestions for performance improvement.

## 2. Proposed Method and Algorithm

The proposed approach relies mainly on generating an atlas by recording the blood pressure responses of an existing mechanistic model for a choice of its component parameters. The employed mechanistic model is detailed in Rosalia et al. [1]. Monte Carlo simulations were run by randomly changing the values of component parameters and recording the resulting blood pressure. This builds an atlas from which to infer the relationship between the chosen component parameters in the mechanistic model and the features extracted from vibroacoustic signals, as well as the relationship between the parameters of the mechanistic model and the resulting blood pressure values, if needed (this means that the mechanistic model can entirely be replaced with a function relating the chosen component parameters to its output blood pressure vector). In the Monte Carlo simulations, parameter values were drawn randomly from the uniform distribution on an interval centered at the corresponding values of that parameter reported in the literature [1] (most of these parameters can be found in Table 1 in [1]).

**Figure 1:**
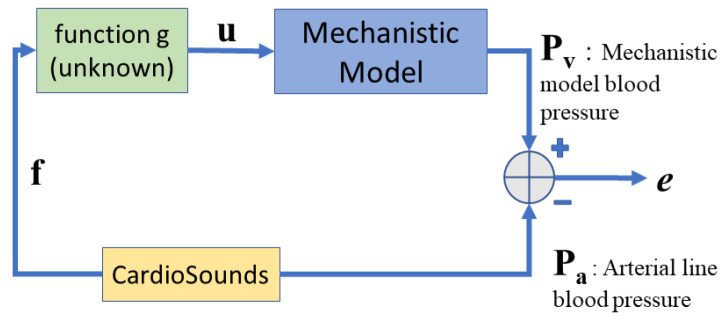
Block diagram of the proposed data-driven approach.

Figure 1 depicts a block diagram of the proposed approach. In this block diagram, the relation between the mechanistic parameter vector u and the vibroacoustic signal feature vector f is denoted by the unknown function *g*, i.e., u=g(f).

**Figure 2:**
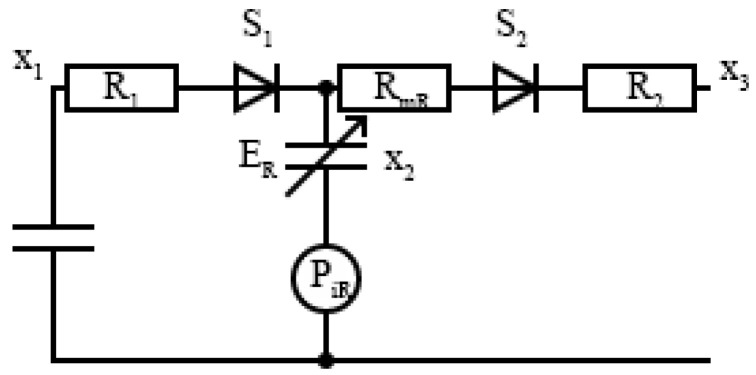
Electronic model of the closed loop cardiovascular system for the right ventricle.

The blood pressure vector $P_{v}$ contains the systolic and diastolic blood pressure values in the steady-state response of the mechanistic model. In the mechanistic model, blood pressure is calculated as a continuous-time variable by solving a system of ordinary differential equations representing the hemodynamics of the cardiovascular system, taken from [6]. This model represents each ventricle at a given time *t* as an isovolumetric pressure generator, $P_{i}\left(t\right)$ that concurrently works with a constant diastolic elastance, $E_{d}$, plus time-varying systolic elastance, $E_{s}$, (i.e., $E\left(t\right)=E_{d}+E_{s}a\left(t\right)$) and a resistance due to myocardium viscosity, $R_{m}$. These two factors help to establish the fluctuation of ventricular pressure based on the volume, V(t), and the rate of volume change, dV/dt. The normalized activation function a(t), representing the time dependence of the isovolumetric pressure, is given by

(1) $$a\left(t\right)=\left\{\begin{array}{cc} 1-cos(2\pi t_{m}/t_{s}), & \mathrm{if}\;t_{m}\;<\;t_{s}\\ 0, & \mathrm{if}\;t_{m}\;>\;t_{s} \end{array}\right.$$

where $t_{s}$ and $t_{c}$ are systolic and cardiac periods defined by $t_{s}=0.16+0.3t_{c}$ and $t_{m}=mod\left(t,t_{c}\right)$, respectively. It follows that the ventricular pressure is given by

(2) $$P_{v}\left(t\right)=\left\{\begin{array}{cc} P_{i}\left(t\right)+E\left(t\right)\times \left(V\left(t\right)-V_{lp}\right)+R_{m}\frac{dV}{dt} & \mathrm{systole}\\ E_{d}\left(V\left(t\right)-V_{lp}\right), & \mathrm{diastole} \end{array}\right.$$

where $V_{lp}$ is the volume at the linearization point and $P_{i}\left(t\right)=P_{i0}a\left(t\right)$ is the isovolumetric pressure at $V_{lp}$ with peak value $P_{i0}$. Therefore, these results can be extended to the right ventricle, $P_{vR}\left(t\right)$, by replacing $P_{i}\left(t\right)$ with $P_{iR}\left(t\right)$, the right peak isovolumetric pressure at volume $V_{lp}$, $R_{m}$ with $R_{mR}$, and E(t) and $E_{d}$ with $E_{R}\left(t\right)$ and $E_{dR}$, respectively. From the analog model shown in Figure 2, it can be seen that the input blood flow from systematic circulation to the right ventricle is regulated by the diodes $S_{1}-S_{2}$ in a series with resistances $R_{1}-R_{3}$, and we can model the tricuspid and pulmonary values, respectively, through

(3) $$\begin{array}{cc} S_{1}\left(t\right) & =\left\{\begin{array}{cc} 1, & \mathrm{if}\;\Delta P_{1}\left(t\right)>0\\ 0, & \mathrm{otherwise} \end{array}\right.\\ S_{2}\left(t\right) & =\left\{\begin{array}{cc} 1, & \mathrm{if}\;\Delta P_{2}\left(t\right)>0\\ 0, & \mathrm{otherwise} \end{array}\right. \end{array}$$

where $\Delta P_{1}\left(t\right)$ and $\Delta P_{2}\left(t\right)$ are functions depending on regulatory functions $P_{iR}\left(t\right)$ and $E_{R}\left(t\right)$, which are related by Kirchhoff’s second law, such that

(4) $$\begin{array}{cc} \Delta P_{1}\left(t\right) & =x_{1}\left(t\right)-P_{iR}\left(t\right)-x_{2}\left(t\right)E_{R}\left(t\right)\\ \Delta P_{2}\left(t\right) & =P_{iR}\left(t\right)+x_{2}\left(t\right)E_{R}\left(t\right)-x_{3}\left(t\right) \end{array}$$

The parameters $x_{1}\left(t\right)$ and $x_{3}\left(t\right)$ are the state variables for the right venous–atrial pressure and pulmonary venous pressure, respectively, while $x_{2}\left(t\right)$ is the right ventricle volume time variation during the systole. Using Ohm’s law, we can define the right ventricular inflow and outflow as

(5) $$\begin{array}{cc} q_{inR}\left(t\right) & =\frac{\Delta P_{1}\left(t\right)S_{1}\left(t\right)}{R_{1}}\\ q_{outR}\left(t\right) & =\frac{\Delta P_{2}\left(t\right)S_{2}\left(t\right)}{R_{mR}+R_{2}} \end{array}$$

The right heart model is completely defined by the right ventricular pressure by applying Kirchhoff’s second law to the electrical model in Figure 2.

(6) $$P_{vR}\left(t\right)=P_{iR}\left(t\right)+E_{R}\left(t\right)x_{2}\left(t\right)-R_{mR}q_{outR}\left(t\right)$$

Likewise, the left heart model can be equivalently derived.

**Figure 3:**
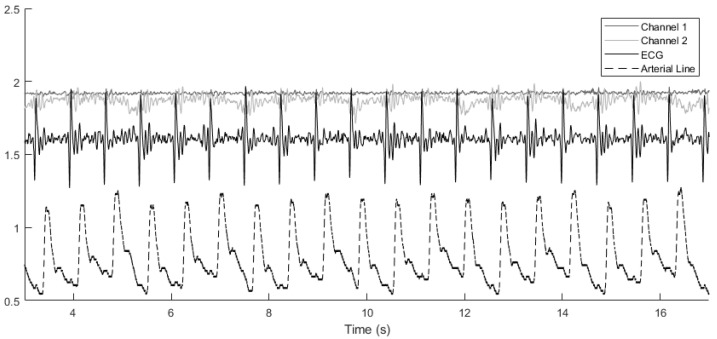
Alignment of various signals across cardiac cycles. The arterial line blood pressure has been scaled down by a factor of 100 for easier comparison. Channel 1 and channel 2 denote the vibroacoustic signals from the sensor.

In our approach, the systolic and diastolic blood pressure values in each cardiac cycle are measured and used in subsequent calculations. Similarly, the ground truth blood pressure vector $P_{a}$ contains the systolic and diastolic values from the arterial line blood pressure continuous-time measurements, as depicted by the red curve in Figure 3. The goal is to learn an unknown function *g* such that the error vector e becomes minimal according to a predefined metric such as the $\ell_{2}$-norm.

For a specific patient, features are extracted from the cardiac cycles in which the vibroacoustic signals are available. To identify the start and end of cardiac cycles, R-peaks detected in an ECG signal included in the vibroacoustic signals are used. To detect R-peaks, the Pan–Tompkins method presented in [7] and implemented in [8] is employed. The proposed algorithm includes the following steps.

1. For each cardiac cycle with vibroacoustic feature vector f, find the $P_{v}$ and corresponding u from the atlas that is closest to $P_{a}$.
2. Repeat across *n* cardiac cycles, i.e., for j=1,…,n, to obtain sets $\left\{u_{j}\right\}$ and $\left\{P_{v,j}\right\}$
3. Learn the function *g* using these sets. Learning candidates considered are a linear model u=Af and a shallow neural network.

The performance of the model can be assessed by calculating the error *e* between $P_{a}$, the ground truth arterial line blood pressure, and $P_{v}$ the blood pressure generated from the mechanistic model using the parameter vector u estimated using f, extracted from held out cardiac cycles and the learned functions *g*.

## 3. Case Study

In this section, real cardiopulmonary acoustic, ECG, and ground truth arterial line blood pressure signals are used to implement and assess the performance of the proposed method when estimating an individual’s blood pressure through the customization of the mechanistic model based on feature extraction from the vibroacoustic sensor.

### 3.1. Vibroacoustic Signals

The vibroacoustic, ECG, and arterial line blood pressure signals were obtained during a retrospective study of hospital inpatients. Data for this analysis were provided by CardioSounds LLC through a data-sharing agreement with the University of Michigan, USA. A vibroacoustic sensor developed by CardioSounds LLC was placed on the patient’s chest to capture non-invasive ECG and heart sound while simultaneous invasive arterial line blood pressure was measured from the patient’s arm. The vibroacoustic signals were recorded on two channels, and therefore two choices of mechanistic component parameters were made. Example signals are plotted in Figure 3, showing how the various signals are aligned with each other across cardiac cycles.

A tenth order low-pass Butterworth filter with a cutoff frequency of 150Hz was used to remove high-frequency noise outside of the frequency band in which heart sounds channels have the majority of their energy content. Next, an eight-level wavelet decomposition was performed using the ‘db4’ wavelet (this wavelet is a member of the Daubechies wavelet family). The detail coefficients of levels four, five, and six were shrunk by soft thresholding where the threshold was selected to minimize Stein’s unbiased risk estimate (SURE) for the entire signal time span [9].

### 3.2. Arterial Line and ECG Signals

For the arterial line blood pressure signal, a low-pass filter with a cutoff frequency of 120Hz was used to remove high-frequency noise. In addition, two notch filters at 60,120Hz were applied to remove power line interference and the first harmonic, followed by a Savitzky–Golay filter of the first order and frame length of 41 to smooth out existing ripples in the data to make reading the systolic and diastolic blood pressure values easier. Similarly, a bandpass filter with lower and higher cutoff frequencies at 0.5Hz and 55Hz, respectively, was applied to the ECG signals.

### 3.3. Choice of Parameters

In the current case, the feature vector f contains two elements from channels 1 and 2, with each element containing the energy in the entire cardiac cycle. Components from the mechanistic model that were expected to represent the heart sounds in a meaningful way were chosen to form the vector u. To this end, for the left ventricle, right ventricle, left atrium, and right atrium, the chosen parameters were the compliance chamber internal diameter and compliance chamber cylindrical chamber length. For the mitral, tricuspid, aortic, and pulmonary valves, the chosen parameters were the laminar flow pressure ratio and minimum area. Therefore, u will be a 16×1 vector containing the above parameters from the mechanistic model. To clarify, the first and second components in u are the left ventricle compliance chamber internal diameter and compliance chamber cylindrical chamber length, respectively. The same pattern is repeated for the right ventricle, left atrium, and right atrium. The ninth and tenth elements in u are the mitral valve laminar flow pressure ratio and minimum area, respectively. Again, the same pattern is repeated for the tricuspid, aortic, and pulmonary valves. Here, the valve minimum area refers to the minimum area of the orifice representing the valve when it is closed. The laminar flow pressure ratio is also a valve parameter that shows if the flow is laminar or turbulent. For more information about the parameters used in u, see [1].

## 4. Simulation and Results

In this section, the simulation results are presented. For each patient, 90 percent of the detected cardiac cycles were used in training, and the remaining cycles were used in testing the performance of the method. The blood pressure was measured at the front end of the descending aorta in the mechanistic model. The systolic and diastolic values were averaged over the last six cardiac cycles in the steady-state response of the mechanistic model. The following metrics for measuring error were considered. The relative error in the norm is defined as

(7) $$e_{r,n}=\frac{{∥P_{v}-P_{a}∥}_{2}}{{∥P_{a}∥}_{2}},$$

and the relative absolute error in either the systolic or diastolic blood pressure values are defined as

(8) $$e_{r,a}=\frac{\left|P_{v}-P_{a}\right|}{P_{a}}$$

where $P_{v}$ represents the blood pressure predicted from the model and $P_{a}$ represents the arterial line ground truth blood pressure value.

Since relative absolute error is presented as a percentage of the ground truth blood pressure value, the acceptable level of error will differ for systolic and diastolic blood pressure. Stage 1 hypertension is categorized as having a systolic blood pressure in the range of 130–139 mm Hg and a diastolic blood pressure in the range of 80–89 mm Hg [10]. Kallioinen et al. performed a systematic review of studies quantifying blood pressure measurement inaccuracy, finding errors at ranges from −23.6 to +33 mm Hg for systolic and −14 to +23 mm Hg for diastolic blood pressure [11]. Therefore, a relative absolute error less than $\frac{33}{130}=0.25$ for systolic and $\frac{23}{80}=0.29$ for diastolic could indicate an improvement in accuracy over traditional methods for blood pressure measurements. Furthermore, since clinical ranges for diagnostic blood pressure categories are incremented by 10 mm Hg, relative absolute errors around $\frac{10}{130}=0.08$ for systolic and $\frac{10}{80}=0.13$ for diastolic would allow for accurate characterization of the general blood pressure level and significant changes therein.

For comparison purposes, a one-layer neural network with ten hidden layers as well as a linear model u=Af were used to represent the function *g*. Data resulting from 5822 Monte Carlo simulations were used to build the atlas. Table 1 lists the range of parameters that were used in simulations, where $u_{i}$ denotes the ith element of u. To make computations faster, the mechanistic model was also replaced by a function approximated by a shallow neural network that was trained using the atlas data (this can be performed using the same sets of component parameter vectors u and blood pressure vectors $P_{v}$ found during training, i.e., $\left\{u_{j}\right\}$ and $\left\{P_{v,j}\right\}$ for j=1,…,n in the algorithm proposed in Section 2), at the cost of introducing error in the resulting blood pressure. The employed neural network had two hidden layers, with ten neurons in the first and five neurons in the second hidden layer.

### Results and Discussion

**Figure 4:**
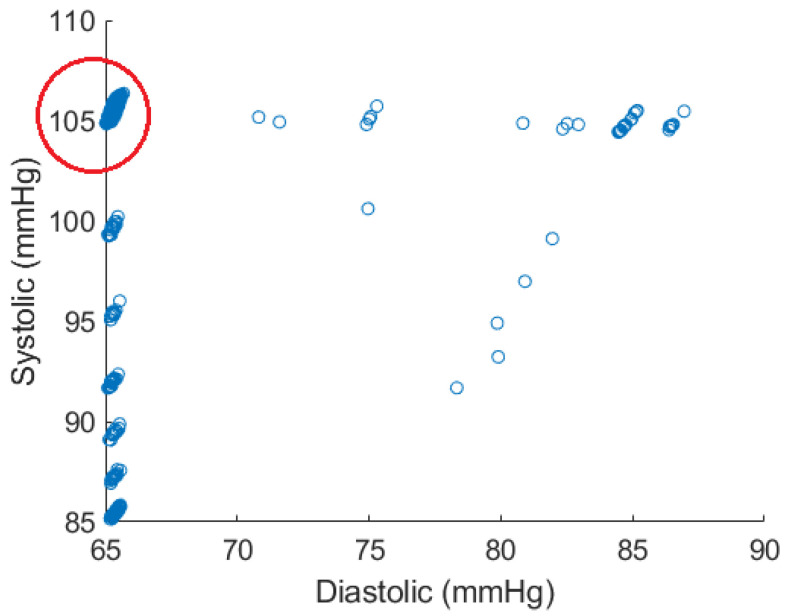
Atlas data points generated by Monte Carlo simulations. The majority of generated points are within the circled region.

Figure 4 shows the distribution of data points in the atlas when the parameter ranges listed in Table 1 are used in the mechanistic model when performing the Monte Carlo trials.

**Figure 5:**
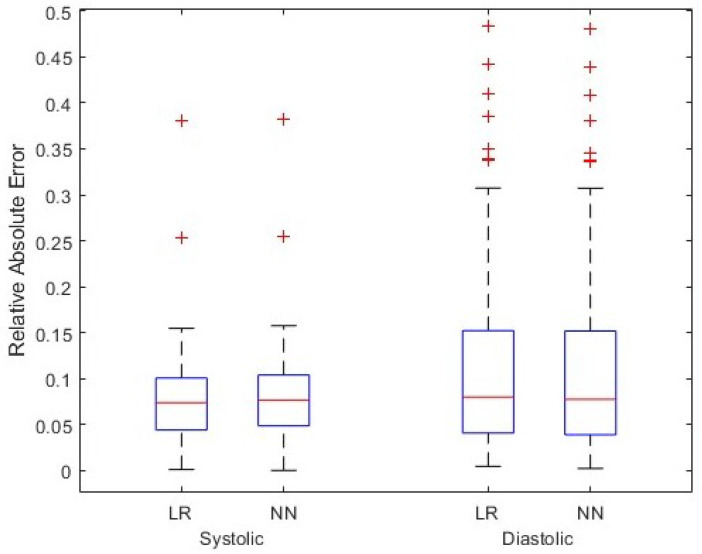
Simulation results for test cardiac cycles across two patients. Two models, linear regression (LR) and shallow neural network (NN), have been assumed for function *g*, and relative absolute error in the systolic and diastolic blood pressure values are shown.

As this figure shows, the spread of data points can be an issue as the points are heavily clustered around certain values. For example, this potentially causes local minima on the error surface on which back propagation is performed when training a neural network. Therefore, the region shown in the red circle was used for training the model. This region contains 5290 data points, constituting the majority of points in the atlas. One also observes that the systolic and diastolic blood pressure values lying in the red circle are tightly clustered around 65 and 105, respectively. Figure 5 shows the results for all test cardiac cycles across two patients. As previously discussed, relative absolute error is expected to be lower for systolic blood pressure, given the larger range in values. Both mean systolic and diastolic relative absolute errors fall below the thresholds previously discussed, suggesting the potential improvement in this method over other non-invasive blood pressure measurement systems. In this small case study, linear regression and the shallow neural network perform similarly, suggesting a linear relationship that can be modeled without complex modeling. However, introducing additional variations and wider blood pressure ranges in larger data sets could require model complexity of the shallow neural network.

Limitations of this approach include the generalizability of assumptions made in the utilized mechanistic model designed by Rosalia et al. [1] across diverse populations. In addition, the atlas resulting from the Monte Carlo trials herein clustered around certain values, which may impact the generalizability of the model when more variations in blood pressure are present. However, it also reduces the risk of overfitting the shallow neural network within the context of the current experiment. It is also worth noting that although the atlas needs to be generated for any choice of parameters forming the vector u, it does not depend on the choice of features extracted from the cardiopulmonary acoustic signals. Therefore, if a set of sufficiently relevant parameters from the mechanistic model is chosen, the atlas needs to be created only once and can be used thereafter for any feature vector. Future work should improve the mechanistic model to remedy the behavior seen in Figure 4 and perform additional simulations to rigorously evaluate this method and its applications in monitoring cardiovascular systems.

## 5. Conclusions

According to the results presented in Section 4, one can conclude that the proposed approach shows promise in integrating a mechanistic model that simulates the hemodynamics of the cardiovascular system and data-driven methods to form a customized blood pressure estimation scheme, especially if the employed mechanistic model is able to generate blood pressure values in a more uniform and diverse range. Also, in this paper, the energy of the entire cardiac cycle in both channels was used to form the feature vector f. To improve results, one can increase the feature space dimension by including other features extracted from segmented heart sounds channels. Example features are time span, frequency, and time–frequency features such as wavelet coefficients.

## Figures and Tables

**Table 1:** Mechanistic model parameters chosen to generate the atlas.

Parameter	Range	Unit
$u_{1}$	3.4–5.4	cm
$u_{2}$	6–8	cm
$u_{3}$	3.8–5.8	cm
$u_{4}$	4–6	cm
$u_{5}$	4–6	cm
$u_{6}$	6.5–8.5	cm
$u_{7}$	4.6–6.6	cm
$u_{8}$	4–6	cm
$u_{9}$	0.9985–0.9995	N/A
$u_{10}$	$0.5\times 10^{-15}$–$1.5\times 10^{-15}$	$m^{2}$
$u_{11}$	0.9985–0.9995	N/A
$u_{12}$	$0.5\times 10^{-15}$–$1.5\times 10^{-15}$	$m^{2}$
$u_{13}$	0.9985–0.9995	N/A
$u_{14}$	$0.5\times 10^{-15}$–$1.5\times 10^{-15}$	$m^{2}$
$u_{15}$	0.9985–0.9995	N/A
$u_{16}$	$0.5\times 10^{-15}$–$1.5\times 10^{-15}$	$m^{2}$

## Data Availability

Data were collected and curated by CardioSounds LLC.
